# The initiation of embryonic-like collagen fibrillogenesis by adult human tendon fibroblasts when cultured under tension

**DOI:** 10.1016/j.biomaterials.2010.02.062

**Published:** 2010-06

**Authors:** Monika L. Bayer, Chin-Yan C. Yeung, Karl E. Kadler, Klaus Qvortrup, Keith Baar, René B. Svensson, S. Peter Magnusson, Michael Krogsgaard, Manuel Koch, Michael Kjaer

**Affiliations:** a Institute of Sports Medicine, Department of Orthopedic Surgery M, Bispebjerg Hospital, and Center for Healthy Aging, Faculty of Health Sciences, University of Copenhagen, 2400 Copenhagen, Denmark; b Wellcome Trust Centre for Cell-Matrix Research, Faculty of Life Sciences, University of Manchester, Manchester, UK; c Biomedical Sciences, Faculty of Health Sciences, University of Copenhagen, Copenhagen, Denmark; d College of Biological Sciences, University of California, Davis, USA; e Department of Orthopedic Surgery M, Bispebjerg Hospital, Copenhagen, Denmark; f Institute for Oral and Muscoskeletal Biology, Center for Biochemistry, Dental School, Medical Faculty, University of Cologne, Cologne, Germany

**Keywords:** Collagen, Extracellular matrix, Fibroblast, Tendon

## Abstract

Tendon fibroblasts synthesize collagen and form fibrils during embryonic development, but to what extent mature fibroblasts are able to recapitulate embryonic development and develop normal tendon structure is unknown. The present study examined the capability of mature human tendon fibroblasts to initiate collagen fibrillogenesis when cultured in fixed-length fibrin gels. Fibroblasts were dissected from semitendinosus and gracilis tendons from healthy humans and cultured in 3D linear fibrin gels. The fibroblasts synthesized an extracellular matrix of parallel collagen fibrils that were aligned along the axis of tension. The fibrils had a homogeneous narrow diameter that was similar to collagen fibrils occurring in embryonic tendon. Immunostaining showed colocalization of collagen type I with collagen III, XII and XIV. A fibronectin network was formed in parallel with the collagen, and fibroblasts stained positive for integrin α_5_. Finally, the presence of cell extensions into the extracellular space with membrane-enclosed fibrils in fibripositors indicated characteristics of embryonic tendon. We conclude that mature human tendon fibroblasts retain an intrinsic capability to perform collagen fibrillogenesis similar to that of developing tendon, which implies that the hormonal/mechanical milieu, rather than intrinsic cellular function, inhibits regenerative potential in mature tendon.

## 1 Introduction

Tendon consists predominantly of collagen type I, and to a lesser degree other fibrillar (type III and V) and non-fibrillar collagens (type XII and XIV), proteoglycans and glycoproteins. In tendons, collagen fibrils are dense, well-organized and aligned in parallel along the main axis of tension [1]. Tendon fibroblasts synthesize collagen during embryonic development and the cells play an active role in the structure formation of the tendon. In particular, aligned collagen fibrils are produced in actin-rich membranous compartments (fibripositors) that extend into the extracellular space suggesting that cells influence matrix assembly in the developing tendon [2–5]. Interestingly, fibripositors have not been reported in postnatal tendon. With increasing age, tendon fibroblasts decrease in number [6,7]. In the mature tendon, tenocytes maintain tendon tissue by synthesizing collagen and collagen-associated proteins [8]. However, it is unknown to what degree the mature tendon fibroblasts are capable of initiating fibrillogenesis and influencing the repair of mature tendon.

Collagen fibril morphology changes during tendon development and maturation. At the onset of embryonic tendon formation, immature fibril intermediates are present with thin uniform fibril diameter and both unipolar and bipolar fibril ends are visible [9,10]. In the mature tendon, collagen fibrils display a larger and more heterogeneous diameter compared to the embryonic state [11]. Unlike in embryonic tendon, mature tendon contains none, or very few fibril ends, suggesting that the fibrils are continuous throughout the entire tendon [12]. This implies that the immature fibrils serve as templates in developing tendon permitting both lateral and linear maturation. However, the potential of the adult human tendon fibroblasts to synthesize immature fibrils *de novo* is not fully elucidated.

During tendon development, linear and lateral growth is a tightly controlled process that involves several molecules. Fibrillar collagen III, and *F*ibril-*A*ssociated *C*ollagens with *I*nterrupted *T*riple Helices (FACITs) XII and XIV have been shown to be involved in the regulation of lateral growth at early stages [13–18]. Other proteins are important for fibrillogenesis and the cell–matrix interaction, such as fibronectin and integrin α_5_β_1_, and collagen assembly has been shown to be dependent upon a fibronectin network and the expression of integrins in animal models [19], for review see [20]. This indicates a coordinated process for tendon formation, but whether adult human tendon cells can regulate fibrillogenesis and the fibril assembly is unknown.

Our purpose was to examine if mature human tendon fibroblasts are capable of regulated collagen fibrillogenesis *ex vivo*. This was done by using a previously described cell–embedded matrix model by Kapacee and collaborators [21].

## 2 Material and methods

### 2.1 Cell origin and processing

Mature human tendon fibroblasts were dissected from the semitendinosus and gracilis tendons of patients undergoing surgery for anterior cruciate ligament (ACL) reconstruction (29 ± 7.5 years). Procedures with human tendon samples were approved by the local ethical committee and informed consent was obtained. Tendon tissue was enzymatically digested overnight in DMEM/F12 (Gibco/Invitrogen) containing 20% fetal calf serum (FCS) (Gibco/Invitrogen) and bacterial collagenase type II (Worthington), and cells collected by centrifugation (600*g*, 6 min). Following two washes with PBS (phosphate-buffered saline, pH 7.3), tendon fibroblasts were seeded in tissue culture flasks and cultured in DMEM/F12, 10% FCS, 100 U/ml penicillin and 100 μg/ml streptomycin (Gibco/Invitrogen). Tendon cells were split no further than the 5th passage and used for experiments in fibrin matrices.

### 2.2 Preparation of culture dishes

Each well of a six-well plate was coated with ∼1.5 ml SYLGARD (Dow-Chemicals) and allowed to set at 55 °C for 48 h. Next, two short silk sutures (0.8 cm, Ethicon) were pinned onto the coated plates with minuitiens insect pins (0.1 mm diameter) (Fine Science Tools GmbH) with a distance of 1.5 cm in between sutures. The plates were sterilized by immersion in 70% ethanol for 45 min.

Human tendon fibroblasts were suspended in 5.0 mg human fibrinogen and 0.44 units of human thrombin (Sigma–Aldrich) to a final concentration of 3.75 × 10^5^ per 750 μl and rapidly spread over the complete surface of the coated wells. The cell-embedded fibrin gel was allowed to set for 30 min at 37 °C, and cultured until the matrix was fully contracted. Every other day, culture medium supplemented with 0.2 mm l-ascorbic acid 2-phosphate (Sigma–Aldrich) was replaced. All constructs in this study were analyzed at Day 0, which was the first timepoint, the constructs formed a continuous tendon-like tissue between the anchors.

### 2.3 Histology

Constructs were embedded in Tissue-Tek and quick-frozen by immersion in isopentane, precooled by liquid nitrogen. Longitudinal sections (8 μm) were cut at −25 °C using a cryostat, mounted on SuperFrost Plus slides (Menzel–Gläser, Braunschweig, Germany), and stored at −80 °C. Sections were analyzed by light and immunofluorescence microscopy. For transmitted light microscopy, samples were stained with Mayers Hematoxylin & Eosin. For fluorescence light microscopy, sections were fixed in 4% paraformaldehyde in PBS for 4 min. Following blocking in 1% bovine serum albumin, 1% skim milk powder in TBS (Tris-buffered saline), the samples were incubated with the respective primary antibodies overnight. After washing, fluorescin-conjugated secondary antibodies (AlexaFluor, Molecular probes) were added and the samples incubated for 60 min at room temperature. This step was followed by a wash and by mounting the sections with ProLong Gold Antifade Reagent with DAPI (4′, 6-diamidino-2-phenylindole) (Invitrogen). Negative controls were incubated identically but without the addition of primary antibodies. The following combinations were performed: Collagen I and collagen III (both Sigma–Aldrich), collagen I and collagen XII (Manuel Koch, affinity purified), collagen I and collagen XIV (Manuel Koch, affinity purified) and fibronectin (Nova Castra) and integrin α_5_ (Santa Cruz Antibodies).

### 2.4 Mechanical tensile testing

Construct integrity was tested on a mechanical test rig during tensile loading. The procedure has been described in detail previously [22]. Briefly, the stage (200-N tensile stage, petri dish version, Deben, Suffolk, UK) consists of a load cell (1% accuracy), a specimen liquid chamber, and two specimen mounting plates driven by a computer-controlled motor fitted with a linear variable differential transducer (LVDT) that registers changes in mounting plate displacement. The mechanical test frame was placed under a stereoscopic microscope with a C-mount lens (×0.38), the microscope was equipped with a 15-Hz digital camera. The silk sutures at the end of the construct were allowed to air dry at room temperature and thereafter glued to the uncoated aluminum end plates of the mechanical rig. The glue was allowed to dry while the mid-portion was kept in saline-soaked gauze. The constructs and end plates were submerged in a petri dish with 0.9% saline solution before loading (0.5 mm/min) the specimen to failure and the resulting force-elongation curves were low-pass filtered by a moving average.

### 2.5 Atomic force microscopy

Tapping mode images of these fibrils were used for visual inspection of D-band periodicity and the presence of fibril ends. Imaging was performed on dry samples using a MultiMode AFM, Nanoscope IIIa controller, J-type scanner and Nanoscope 6.0 software (Vecco Instruments) with Olympus OMCL-AC160TS cantilevers.

### 2.6 Transmission electron microscopy

After discarding culture medium and a rinse in PBS, constructs were fixed by immersion in 2% glutaraldehyde in 0.05 m sodium phosphate buffer (pH 7.2) and stored cold at 4 °C. Following fixation, specimens were dehydrated in graded series of ethanol, transferred to propylene oxide and embedded in Epon (Hexicon, Houston, Texas, USA) according to standard procedures. Ultrathin sections were cut both transversely and longitudinally with a Reichert–Jung Ultracut E microtome and collected on one hole copper grids with Formvar supporting membranes, and stained with uranyl acetate and lead citrate.

For negative stain analysis, constructs were homogenized with a pestle in 500 μl 0.15 m sodium phosphate buffer (pH 7.2). Fine suspensions of the material were negatively stained with 2% potassium phosphotungstate (pH 7.0) on 200 mesh copper grids with Formvar supporting membranes. The sections and negatively stained specimens were examined with a Philips CM 100 transmission electron microscopy (TEM) operated at an accelerating voltage of 80 kV. Images were recorded with a Megaview 2 camera and processes with the Analysis software package.

The NIH-based image-processing program, Image J, was used for measurement of collagen fibril diameter. On randomly selected transverse micrographs, 700 fibrils were analyzed in total. Data is expressed in nm as mean ± S.E.M.

## 3 Results

### 3.1 Formation of tendon constructs

**Fig. 1:**
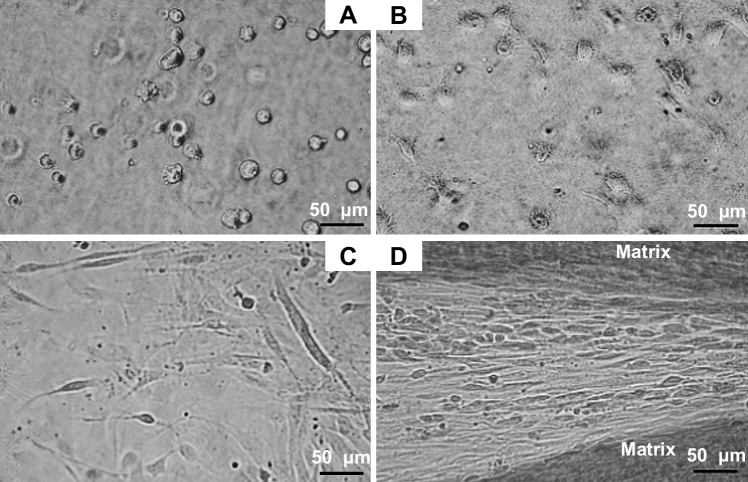
Human tendon fibroblasts embedded in a 3D fibrin gel. (A) Tendon fibroblasts display a rounded cell morphology shortly after embedding in fibrin. The image is taken approximately 15 min following cell seeding. Bar: 50 μm. (B) Fibroblasts are expanding and form cellular extensions 180 min following seeding. Bar: 50 μm. (C) Fibroblasts display a thin, elongated cell body and long extensions, the random alignment is seen in the early phase (first three days) of construct formation. Bar: 50 μm. (D) Parallel arrangement of tendon fibroblasts, which are located in between a synthesized matrix with high cellularity. The ordered alignment is visible during the late stage of construct contraction and tissue formation. Bar: 50 μm.

The 3D matrix constructs were inspected every second day by phase contrast microscopy. The mature human tendon fibroblasts contracted the fibrin matrix over a period of eight to ten days until the initial circular fibrin matrix formed a continuous tendon-like tissue between the sutures. Initially, the cells showed a rounded morphology, but began to form cellular extensions and spread out within hours following seeding (Fig. 1A and B). On the days following embedding, the tendon fibroblasts displayed an elongated morphology with long cellular extensions. The cellular arrangement in the early phase, i.e. the first 3 days, of construct formation was rather random and appeared less ordered (Fig. 1C). During matrix formation, the fibroblasts became organized in a parallel arrangement (Fig. 1D). The gradual matrix contraction was a reproducible process, in case of failure to form tendon constructs, the matrix detached from the SYLGARD layer within the first two days. This occurred in approximately 20% of the cases.

### 3.2 Histological evaluation of tendon constructs

**Fig. 2:**
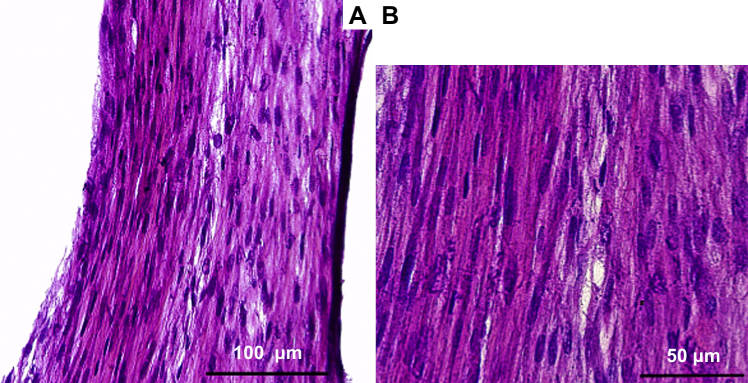
H&E staining of a longitudinal cryosection of a tendon construct. Parallel alignment of fibrils in the extracellular matrix and the dense arrangement of cell nuclei are demonstrated. Bar 2A: 100 μm, Bar 2B: 50 μm.

**Fig. 3:**
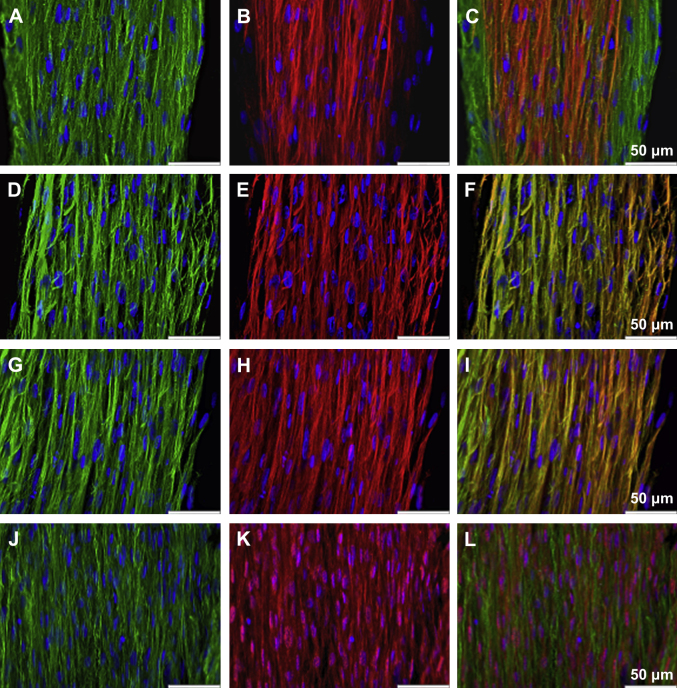
Immunohistological images of tendon constructs. (A) Collagen I (green) and nuclear counterstain DAPI (blue), (B) collagen III (red) and DAPI (blue), (C) computer-generated merged images of the individually captured images. Bar: 50 μm, (D) Collagen I (green) and DAPI (blue), (E) collagen XII (red) and DAPI (blue), (F) computer-generated merged images of the individually captured images. Bar: 50 μm, (G) Collagen I (green) and DAPI (blue), (H) collagen XIV (red) and DAPI (blue), (I) computer-generated merged images of the individually captured images. Bar: 50 μm, (J) Fibronectin (green) and DAPI (blue) (K) integrin α5 (red) and DAPI (blue), (L) computer-generated merged images of the individually captured images. Bar: 50 μm.

The parallel arrangement of collagen fibrils and of fibroblasts is characteristic of tendon tissue. Fig. 2 shows the alignment of the ECM (ExtraCellular Matrix) structure and the cellular arrangement within the construct. From this image, it can be seen that the cell density in the tendon constructs is high. Immunolocalization using antibodies specific for type I collagen show that the cells had synthesized an ECM rich in tendon-like collagen fibrils (Fig. 3A, D and G). By means of immunohistology, a colocalization of collagen type I with the fibrillar collagen III is found in the midsection of the tendon construct. As seen in the figure, collagen III is not expressed in the outer areas of the tendon constructs at this timepoint (Fig. 3A–C).

Binding of the FACITs collagen XII and collagen XIV with collagen I has been described during embryonic tendon development. The spatial shift of collagen XII and XIV to the endotendinium during maturation is a characteristic of development and the homogeneous distribution of collagen XII and XIV co-localized with collagen I in the tendon proper has not been detected in mature tendon. As visualized in Fig. 3D–F and Fig. 3G–I, collagen XII and collagen XIV is found throughout the constructs, the expression is homogeneously distributed and both collagen XII and collagen XIV are co-localized with type I collagen fibrils in the ECM.

*In vivo*, fibronectin and integrins within the tendon cells are essential for ECM synthesis and collagen fibril assembly. Throughout the constructs, a regular and parallel fibronectin network was found, as shown in Fig. 3J–L, and the cells expressed integrin α_5._

### 3.3 Electron microscopy of tendon constructs

**Fig. 4:**
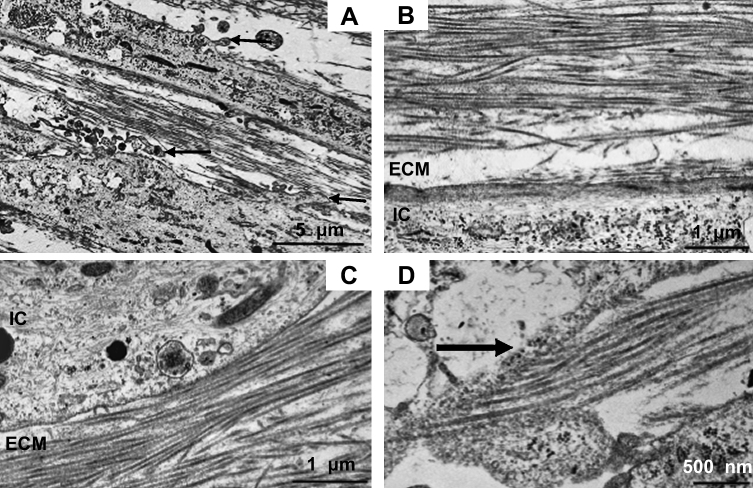
Transmission electron micrographs of tendon constructs. (A)–(D) longitudinally sectioned micrographs. (A) The image shows the arrangement of collagen fibrils in between two tendon fibroblasts. Arrows point to membrane convolutions. Bar: 5 μm. (B) Parallel alignment of collagen fibrils in the ECM adjacent to a tendon fibroblast. Bar: 1 μm. (C) The electron micrograph displays collagen fibrils in the extracellular space in close proximity to a tendon fibroblast. Bar: 1 μm. (D) Collagen-deposition site on a fibroblast is shown. Bar: 500 nm ECM: extracellular matrix, IC: intracellular space.

The structural alignment of collagen fibrils was examined by TEM on longitudinal sections of the constructs. On the cellular level, the shape of the plasma membrane, intracellular filaments and cellular organelles were inspected on micrographs. Fig. 4A depicts the arrangement of collagen fibrils between two tendon fibroblasts and further illustrates the presence of plasma membrane convolutions (arrows). The ordered organization of newly synthesized collagen fibrils in parallel adjacent to a tendon fibroblast is shown in Fig. 4B and C.

The development of a collagen-rich ECM with the parallel alignment reported in tendons, is dependent on cellular processes during tissue morphogenesis. Fig. 4D displays a collagen fibril deposition site and from this caption, the parallel orientation of secreted fibrils is shown. The homogeneous nature of the collagen fibrils in term of diameter is visible in all images of Fig. 4.

**Fig. 5:**
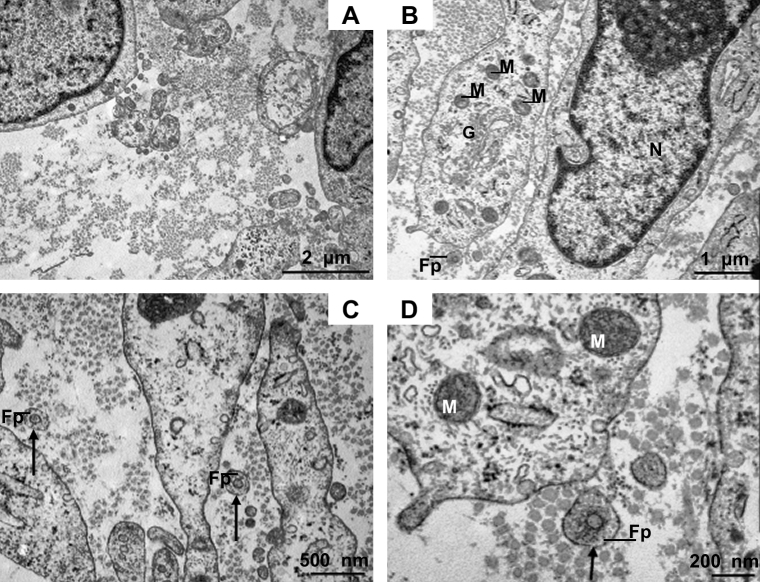
Transmission electron micrographs of tendon constructs. (A)–(D) transverse sectioned micrographs. (A) The image demonstrates the uniformity of collagen fibrils with a narrow diameter. Bar: 2 μm. (B) Tendon fibroblasts in close proximity and collagen fibrils in the extracellular space. In the intracellular space, mitochondria, the Golgi apparatus and the nucleus can be identified. Bar: 1 μm. (C) Collagen fibrils in the extracellular space are visible and collagen fibrils within fibripositors clearly identifiable. Fibripositors are indicated by arrows. Bar: 500 nm. (D) Collagen fibril in fibripositors at high magnification. Bar: 200 nm. G: Golgi apparatus, Fp: fibripositors, M: mitochondrion, N: nucleus.

Fig. 5 contains images of transversely cut electron micrographs. The homogeneous distribution of collagen fibrils narrow in diameter is visible. Fibril diameter of randomly chosen captions was measured and calculated (42.1 ± 0.2 nm, *n* = 700). Also on the transverse plane, multiple membrane extensions can be identified.

Canty and collaborators [3] have identified collagen fibrils within actin-rich fibripositors in embryonic tendon, but fibripositors have not been found in postnatal tendon. Here we show that tendon fibroblasts derived from human adult tissue form fibripositors in the constructs. Fig. 5C and D displays the appearance of collagen fibrils with a narrow diameter in fibripositors (indicated by arrows).

**Fig. 6:**
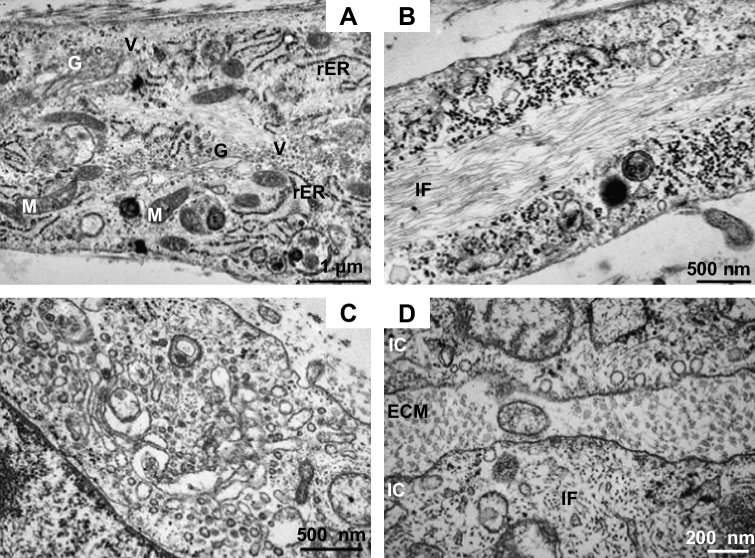
Electron micrographs of tendon fibroblasts, longitudinally (A, B) and transverse (C, D) sectioned. (A) The electron micrograph displays the intracellular space with cell organelles. The Golgi apparatus is seen in close proximity of multiple vesicles, the cell has formed an extensive network of rough Endoplasmatic Reticulum. Several mitochondria are visible. Bar: 1 μm. (B) Image shows a micrograph with an elaborate regular parallel arrangement of intracellular filaments. Bar: 500 nm. (C) Golgi apparatus with multiple vesicles at high magnification. Bar: 500 nm. (D) The transverse section displays the extensive network of intracellular filaments. Bar: 200 nm. G: Golgi apparatus, IF: intracellular filaments, M: mitochondrion, rER: rough endoplasmatic reticulum, V: vesicle.

Longitudinally sectioned micrographs of tendon fibroblasts within the construct are shown in Fig. 6A and B. The presence of an elaborate network of rough endoplasmatic reticulum (rER) and the Golgi apparatus with multiple vesicles (Fig. 6A) displays cell organelles indicative of high synthetic activity, active protein sorting, shuffling and secretion. The Golgi apparatus with numerous vesicles in proximity of the cell organelle is visualized in Fig. 6C. An extended network of intracellular filaments within the tendon fibroblasts is shown in Fig. 6B. The parallel aligned intracellular filaments were found in all the cells inspected by electron micrographs. Fig. 6D displays the filamentous network on a transverse plane.

**Fig. 7:**
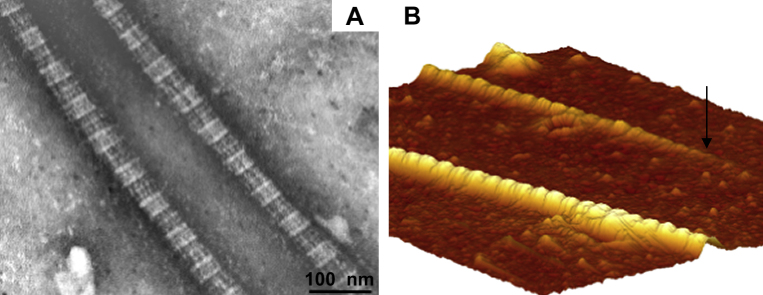
Electron micrograph and image obtained by Atomic Force Microscopy. (A) The image shows two negatively stained collagen fibrils, demonstrating the regular banding pattern of collagen. Bar: 100 nm (B) 3D reconstruction of an atomic force micrograph, which reveals the characteristic collagen banding pattern and shows a collagen fibril end (indicated by arrow). The original image in 2D has a size of 2 μm × 2 μm.

The 67 nm periodicity is a characteristic of fibrillar collagen. The regular banding pattern of negatively stained collagen fibrils depicted in Fig. 7A is in agreement with collagen fibrils visualized by AFM (Fig. 7B). Fig. 7B not only displays the banding pattern, but also the presence of collagen fibrils ends (indicated by arrow).

### 3.4 Tensile testing of the constructs

**Fig. 8:**
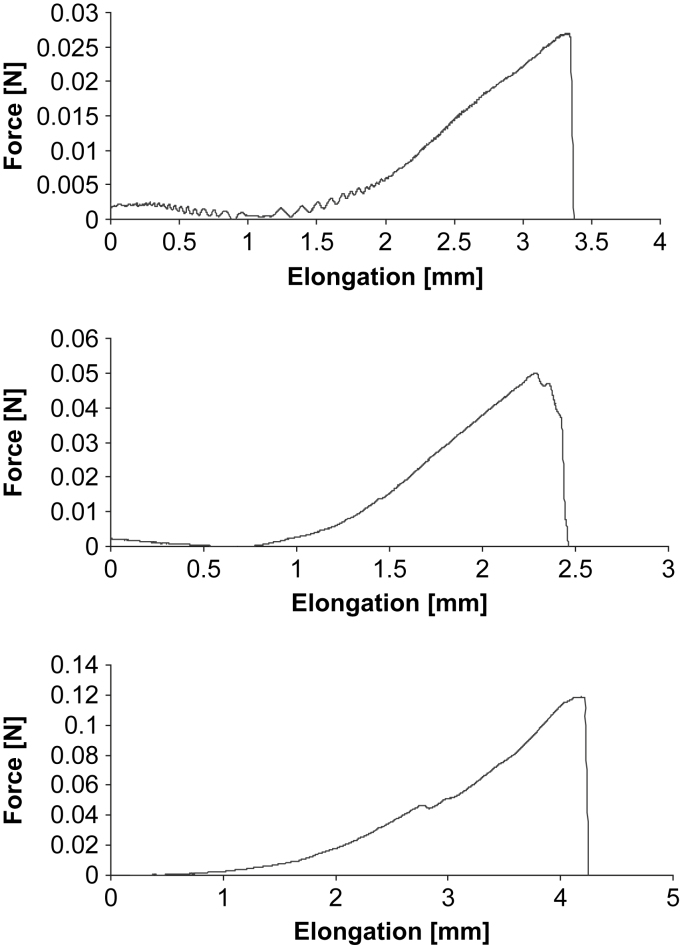
Force-elongation curve of a tendon construct pulled until failure, raw data of three experiments. The curves show the well-described toe and linear region and serves as a test of the tendon constructs’ integrity and mechanical behavior.

These experiments served as a test of the mechanical integrity and function of the constructs. Fig. 8 displays the force-elongation curves of three constructs. Mechanical testing yielded characteristic mechanical behavior associated with force-deformation of tendon tissue [23]. There was a toe-region with a relatively large deformation relative to small changes in applied force, which was follow by a subsequent steeper linear slope of the force-deformation curve.

## 4 Discussion

Mature human tendon fibroblasts embedded in a fibrin gel remodel the matrix to form a linear tendon construct within ten days. The cellular network becomes organized in a parallel arrangement concomitant with matrix contraction and with the synthesis and secretion of collagen fibrils with a uniform, narrow diameter (Figs. 4, 5 and 7). In the constructs, the fibroblasts are in close proximity to each other, the plasma membranes are extensively convoluted and collagen fibrils in fibripositors have been identified (Fig. 5). The collagen fibrils are aligned along the axis of tension, and we found a colocalization of collagen I with collagen III, XII, and XIV (Fig. 3A–I). Furthermore, a dense network of fibronectin was found and fibroblasts stained positively for integrin α_5_, suggesting cell–matrix interactions with numerous adhesion structures (Fig. 3J–L). Our findings strongly suggest that adult tendon fibroblasts in a 3D matrix are actively involved in the *de novo* arrangement of collagen fibrils in the extracellular space and are capable of recapitulating the fibrillogenesis of the developing tendon.

The fact that mature human tendon fibroblasts were found to synthesize collagen fibrils with a homogeneous and narrow diameter (Figs. 4 and 5) was surprising, since this was thought to be a behavior seen only from embryonic tendon cells. On the other hand, the findings match very well with observations in rat tendon cells obtained from postnatal tissue [24] and more recently with observations from human fibroblasts in culture [25]. The latter publication examined human fibroblasts from dermis and tendon and demonstrated the formation of fibrils with a narrow-sized diameter in a cell culture system studied 5–18 weeks after cells were embedded in a matrix engineered from polyglyconic acid. *In vivo*, Postacchini and Martino [26] described the formation of thin immature collagen fibrils in rabbit calcaneal tendon following partial tenotomy with subsequent maturation of fibrils.

In the current study, the constructs were analyzed at day 0, i.e. the day when a continuous linear matrix was formed, and the timepoint might explain the short fibrils with a narrow diameter (42.1 ± 0.2 nm), the presence of cell extensions into the extracellular space, collagen fibrils in fibripositors, and the pronounced convolution of the plasma membrane of tendon fibroblasts, which are all characteristics of tendon during development. In mature tendon, these characteristics have not been found. The distribution and localization of collagen III, XII and XIV also represent processes during tendon development. The distribution pattern of collagen III and XII demonstrates a spatial shift during development [15,27]. The early phase is characterized by a homogeneous colocalization of collagen III and XII with collagen I fibrils in the tendon proper, in later stages, the localization of the collagen types have only been found in the tendon sheath [11,15,16,27]. Histological analysis of the constructs showed clearly a colocalization of collagen I and III in the midsection of the constructs, and of type I and type XII throughout the matrix. Collagen XIV has been shown to be inhibiting to lateral growth of collagen I fibrils [13] and we found a strong immunoreactivity of collagen XIV with collagen I fibrils suggesting that this process is maintained in 3D culture. Collagen XIV binding to type I collagen fibrils matches to the presence of immature fibrils with a narrow diameter.

The assembly of the collagen and fibronectin network as well as integrin expression is tightly regulated during development [19,20,28,29]. We found the establishment of a collagen I rich matrix concomitant with fibronectin assembly and integrin expression throughout the tendon construct, which suggests that the cells sense the tension developed through anchoring points in this 3D culture model and translate the stimuli into intracellular signals. Not only is information transmitted through integrin receptors from the ECM into the cells, but also from the intracellular space to the ECM and this interplay most likely has an essential role in expression and assembly of ECM components [19,28,30,31], for review see [20,32]. Although integrin activation and associated signaling pathways were not investigated in the present study, the findings suggest that tendon fibroblasts rapidly synthesize an ECM, to which the cells become linked through adhesion structures. The finding of pronounced integrin α_5_ expression in the tendon constructs indicates the establishment of focal adhesions, which might be involved in the development of a parallel fibrillar network of collagen.

We suggest that the experimental conditions in this study had a stimulating effect on the tendon fibroblasts to initiate processing and deposition of collagen fibrils that shares features of immature tendon. Starting with a high cell-to-ECM and a high cell-to-cell ratio might be potent activators of developmental tendon fibrillogenesis. This hypothesis supports the present finding that mature tendon fibroblasts have the intrinsic capacity to behave like cells in the developing tendon, and suggests that the environment in developing and mature tendon determines mechanisms of fibril synthesis, deposition and alignment.

A high cell number could stimulate embryonic fibrillogenesis. As seen in Figs. 1–3, there is a high cell-to-matrix ratio in the present study, in contrast to cell–matrix ratio seen in mature human tendon [6]. Cell–cell junctions allow cells to establish cell polarity and guide the cellular responses to the local environment [33,34]. Richardson and co-workers [5] found that the structural integrity of the developing tendon in the embryo is dependent on cell condensation through cell–cell interactions mediated by the junctional protein cadherin-11. A loss of cell contacts resulted in a concomitant loss of structural alignment of collagen fibrils. Also in the mature tendon, fibroblasts demonstrate immunoreactivity for cadherin-11 *in vivo* (Bayer ML, unpublished observation) and isolated human tendon fibroblasts retain the ability to form cadherin-11 mediated cell junctions in 2D [35]. This indicates that tendon fibroblasts form multiple cell junctions in the tissue, but also when isolated from the natural environment. In the tendon constructs, we found high immunoreactivity for cadherin-11 and connexin43 (data not shown), which suggests that the cells are interconnected and actively interacting during matrix formation.

Culture conditions might have a substantial impact on the results presented here. Fibrin is a substrate which is used extensively in tissue engineering [36], but it is not known, whether the human mature tendon fibroblasts respond with an injury response to the fibrin, given its natural occurrence during wound healing, and this reaction would explain the immature narrow-sized collagen fibrils. However, Deng and collaborators [25] reported narrow-size fibril diameter in the range of ∼20 nm after 5–9 weeks culturing in a 3D matrix made of polyglycolic acid. This suggests that the results seen in our study are not a direct response to fibrin. Postacchini and Martino [26] described the synthesis of narrow-sized fibrils in an animal model following tendon injury. This might appear as an injury response, however, the fibrils underwent maturation in the weeks following injury, which indicates that collagen fibrillogenesis begins with immature thin collagen fibrils that subsequently mature. Moreover, it appears as if the fibrillogenesis we report is a well-ordered process. The appearance of collagen-associated proteins, the fibronectin network and the development of fibripositors are considered as support for this assumption. Further, we report the characteristic toe and linear region of the force-elongation curve when constructs were mechanically tested (Fig. 8). Accordingly, our findings are in contrast to what would be expected in unstructured scar formation after injury.

The choice, amount and composition of the serum have effects on cell proliferation, ECM production and force production of engineered tissue [37,38]. Earlier studies on tendon and muscle engineering have applied a reduction from high to low serum content when cells in the model reached confluency [24,38], whereas other researcher have not altered serum concentration throughout the culturing period [21,25]. In the present study, we chose not to change serum concentration over the culturing period. It cannot be excluded that some of the findings in collagen fibrillogenesis were triggered by creating a somewhat *fetal* environment by the supplementation of a multitude of growth factors in high amounts throughout construct formation. Even if this is so, the findings demonstrate the capacity of fibroblasts to recapitulate embryonic mechanisms in a proper environment. This is in line with the idea that the hormonal and mechanical environment rather than the intrinsic cell activity determines cell behavior. This fits with findings in both animal and human skeletal muscle satellite cells, where isolated cells in culture demonstrated a behavior according to the characteristics of the growing medium, and where older cells showed rejuvenation when subjected to serum from young donors [39,40]. This view is interesting from a clinical perspective, as it is well-described that mature human tendon has a poor regenerative capacity [35]. Potentially, the regenerative problem may not relate to any intrinsic cell deficiency, but rather relates to an unfavorable environment, that does not allow for collagen fibrillogenesis.

## 5 Conclusion

We report that fibroblasts derived from mature human tendon synthesize a collagen matrix that is characterized by fibrils with a homogeneous narrow-sized diameter aligned in parallel, by the presence of fibril ends and the occurrence of collagen fibrils enclosed within a membrane in the extracellular space. In addition, the pattern of molecules associated with collagen type I resembles the finding in developing tendon. Therefore, the results indicate that mature human tendon fibroblasts retain an intrinsic capability of performing collagen fibrillogenesis similar to developing tendon. This implies that the environment rather than the cells inhibit an effective regenerative potential in mature tendon tissue.

## Appendix

Figures with essential colour discrimination. Figs. 2, 4, 5 and 7 in this article are difficult to interpret in black and white. The full colour images can be found in the on-line version, at doi:10.1016/j.biomaterials.2010.02.062.
